# Foreword

**DOI:** 10.19102/icrm.2021.120126S

**Published:** 2021-01-15

**Authors:** Ashit G. Patel


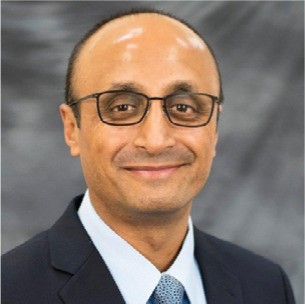


Dear Readers,

The 12-lead electrocardiogram remains the gold standard in three-dimensional mapping of abnormal cardiac electrical signatures and is essential for constructing a preprocedural blueprint. Acquired through the manipulation of electrode-fitted catheters, the electrogram (EGM) is also a fundamental component supporting the diagnosis, mapping, and treatment of cardiac arrhythmias. Visualized and recorded in many ways, yet having its own limitations, the traditionally filtered bipolar EGM is most commonly used. Though bipolar recording limitations such as directionality and contact force influence are universally recognized, they have been significantly lessened with the emergence of the Advisor™ HD Grid Mapping Catheter, Sensor Enabled™ (Abbott, Chicago, IL, USA).

The Advisor™ HD Grid catheter is an 18-electrode, four-by-four electrode–compliant splined configuration with an additional bipole located on the shaft for nonfluoroscopic use. This configuration creates a mesh that allows for up to 32 orthogonally oriented recordings to be created. Moreover, the catheter’s unique high-density grid design provides solutions for the two most commonly encountered issues in electroanatomic mapping. First is directionality or “bipolar blindness,” which is historically well described and studied, such as by Tung et al. in 2016.^1^ Initially, with the inception of bipolar EGM technology, paired with the surrendering of unipolar EGM, directionality limitations have become widely scrutinized. Ndrepepa et al. previously helped to demonstrate bipolar and unipolar EGM limitations and value in 1995.^2^ High-density grid technology has the ability to facilitate novel techniques such as omnipolar mapping as described by Massé et al.,^3^ who concluded that electrode orientation-independent cardiac wavefront trajectory and speed at a single location can be determined with omnipolar EGMs. Other possibilities and future applications could include the collection of four-dimensional information.

The second issue, diagnostic catheter contact assessment, relates to the effects of contact force on electrogram voltage and has been discussed by Mizuno et al.,^4^ who showed in 2013 that, with varying degrees of measured force, a proportionate discrepancy in the bipolar EGM recording was apparent. The Advisor™ HD Grid technology has shown the ability to visualize tissue contact based on spline perturbation. Different degrees of perturbance are associated with the results of a rudimentary test measured in grams of force. Grid angulation at 45 degrees equates to 5 g of force, while that at 90 degrees equates to 10 g of force. Visualization of this angulation can also be helpful in preventing complications such as perforation.

Though the ability to localize and reconstruct electrical and physical anatomy safely, accurately, and efficiently is greatly enhanced with the existing knowledge, further studies to show additional uses and future applications are still required. The following supplement includes a compendium of experiences from electrophysiologists who have implemented the Advisor™ HD Grid catheter into their clinical practice.

Sincerely,

Ashit G. Patel, md, facc, fhrs

Cardiac Electrophysiologist

Cascade Cardiology, LLC

Salem, OR, USA
